# Circular RNA hsa_Circ_0007552 inhibits lung adenocarcinoma proliferation, migration and invasion via the miR-7974/BAP1 axis

**DOI:** 10.3389/fimmu.2025.1634326

**Published:** 2025-08-21

**Authors:** Yanping Lin, Rong Li, Xin Shen, Mingjin Wu, Jiadai Tang, Jiangyan Guo, Caixin Yin, Tingrong Xia, Dongqi Li, Fengdi Hu, Yedan Liao, Rui Li, Lin Xie

**Affiliations:** ^1^ Department of Digestive Oncology, The Third Affiliated Hospital of Kunming Medical University, Yunnan Cancer Hospital, Peking University Cancer Hospital Yunnan, Kunming, Yunnan, China; ^2^ Kunming Medical University, Kunming, Yunnan, China; ^3^ Department of Pathology, The Third Affiliated Hospital of Kunming Medical University, Yunnan Cancer Hospital, Peking University Cancer Hospital Yunnan, Kunming, Yunnan, China; ^4^ Bone and Soft Tissue Tumors Research Center of Yunnan Province, Department of Orthopaedics, The Third Affiliated Hospital of Kunming Medical University, Yunnan Cancer Hospital, Peking University Cancer Hospital Yunnan, Kunming, Yunnan, China

**Keywords:** LUAD, Circ_0007552, miR-7974, BAP1, migration

## Abstract

**Objectives:**

Circular RNAs (circRNAs) are a class of non-coding RNAs with diverse pathophysiological functions. However, the functional roles and molecular mechanisms of circRNAs in lung adenocarcinoma (LUAD) remain to be further elucidated.

**Methods:**

The expression levels of Circ_0007552 (Circ_RILPL1), miR-7974, and BRCA1-associated protein 1 (BAP1) mRNA in LUAD tissues and cells were detected by quantitative real-time PCR (qRT-PCR). Cell viability, migration, and invasion capabilities were evaluated using the Cell Counting Kit-8 (CCK-8), colony formation assay, wound healing assay, and Transwell migration and invasion assays. A xenograft tumor model in nude mice was established to assess the *in vivo* effects of Circ_0007552 on LUAD by measuring tumor size, weight, and growth rate. Bioinformatics analysis and dual-luciferase reporter gene assays were conducted to validate the interactions among Circ_0007552, miR-7974, and BAP1. Western blot was performed to detect the protein expression of BAP1.

**Results:**

Circ_0007552 exhibited low expression in LUAD tissues and cells, correlating with clinicopathological features such as tumor size, lymph node metastasis, clinical stage, and poor prognosis. Both *in vitro* and *in vivo* experiments demonstrated that Circ_0007552 overexpression suppressed malignant biological behaviors of LUAD cells, whereas its knockdown exerted opposite effects. Mechanistically, Circ_0007552 functioned as a competing endogenous RNA (ceRNA) for miR-7974, negatively regulating its expression. Overexpression of miR-7974 partially reversed the tumor-suppressive effects of Circ_0007552 on LUAD cells. Furthermore, BAP1 was identified as a downstream target of miR-7974, and Circ_0007552 positively modulated BAP1 expression.

**Conclusion:**

Circ_0007552 inhibits the development, progression, and metastasis of lung cancer by sponging miR-7974 to upregulate BAP1 expression. The present study further elucidates the underlying molecular mechanisms driving lung cancer progression.

## Introduction

Lung cancer is the leading cause of tumor-related deaths globally, responsible for 18.4% of cancer fatalities (1). Lung adenocarcinoma, the most prevalent subtype, constitutes 50% of lung cancer cases, with its incidence rising annually. It is characterized by poor prognosis, low survival rates, and a tendency for distant metastasis, particularly to bone. The 2-year survival rate for patients with bone metastasis from lung adenocarcinoma is under 11.3%, with an average survival of just 9.7 months (2). Bone metastasis causes persistent pain, significantly diminishing quality of life, and heightening the risk of complications like hypercalcemia, pathological fractures, spinal cord compression, and paraplegia (3). Thus, effective strategies to inhibit bone metastasis remain an urgent medical challenge. Currently, there are no reliable methods for early diagnosis or effective prevention and treatment for bone metastasis in lung adenocarcinoma patients. Standard treatments such as chemotherapy, radiotherapy, and immunotherapy do not effectively target bone metastasis, while surgical options are limited. Although drugs like strontium-89, bisphosphonates, and denosumab(AMC-162) show some efficacy, their long-term use can lead to complications, and their therapeutic effects are not sustained (4). Therefore, identifying new molecular markers for lung adenocarcinoma metastasis and developing innovative prognostic assessment methods are critically important for clinical practice.

In recent years, our research group has conducted studies on the roles and mechanisms of ncRNAs in tumor metastasis. We examined the expression and regulatory networks of ncRNAs and miRNAs in metastatic tumors, analyzing their functions and mechanisms (5). Additionally, we investigated the interactions between circRNAs and miRNAs, highlighting circRNAs’ roles in signal transduction pathways and their potential as clinical biomarkers (6). In our previous study (7), A total of 2141 differentially expressed RNAs were screened by whole transcriptome sequencing from lung adenocarcinoma tissue and bone metastasis tissue samples, among which 706 circular RNAs and 43 miRNAs were differentially expressed. Circ_0007552 was significantly down-regulated. To further investigate the potential regulatory mechanism of Circ_0007552 on lung adenocarcinoma metastasis, differential analysis of the ceRNA network and sequencing database comparisons suggest that Circ_0007552 may function as a ceRNA by influencing lung adenocarcinoma metastasis through miR-7974 (7). Using miRanda, we analyzed miR-7974 binding circRNAs and found five differentially expressed circRNA relationships. Among these, Circ_0007552 and Circ_0005039 were negatively regulated by miR-7974, with Circ_0007552 showing low expression in our sequencing data.

## Materials and methods

### Clinic tissue samples

Tissue specimens for this study were obtained from lung adenocarcinoma patients at the Third Affiliated Hospital of Kunming Medical University, with ethics committee approval. Fresh primary lung adenocarcinoma tissues, paracancerous tissues located over 5 cm from the tumor margin, and bone metastasis tissues were collected from patients admitted between March and May 2019. Each specimen was immersed in 1 ml of RNA later in cryopreservation tubes and transported to a 4°C refrigerator within 30 minutes, then stored overnight at -80°C. We collected 30 pairs of primary lung adenocarcinoma tissues and their corresponding paracancerous tissues from non-metastatic patients, along with 20 samples of bone metastasis tissues. Patient data, including gender, age, ethnicity, smoking history, tumor size, TNM stage, lymph node metastasis status, and survival time, was also recorded. This experiment was carried out under the approval and guidance of the Ethics Committee of Yunnan Cancer Hospital, the third Affiliated Hospital of Kunming Medical University (approval number: KYLX202183).

### Cell culture

Normal human bronchial epithelial cells (BEAS-2B) and human LUAD cell lines A549, PC-9, SPCA1, H1299, H838, H1734, and 95-D were sourced from the Yunnan Cancer Institute. BEAS-2B cells were cultured in DMEM (Gibco, Grand Island, NY), while the LUAD cell lines were maintained in RPMI-1640 (Biosharp) with 10% fetal bovine serum (Gibco, Grand Island, NY) at 37°C and 5% CO2 under saturated humidity.

### Fluorescence *in situ* hybridization

The biotin-labeled Circ-0007552 probe was synthesized, and related kits were acquired from GenePharma Corporation (Shanghai, China), with probe sequences listed in **Supplementary Table S9**. In fluorescence *in situ* hybridization experiments, Ten thousand cells of H1299 and SPCA1 were seeded onto cell slides in a 48-well plate and cultured for 18 hours. Then fixed with 4% paraformaldehyde (15 min, RT) after PBS washing. Following permeabilization with 0.1% Buffer A (15 min, RT) and blocking (37°C, 30 min), cells were equilibrated in 2×Buffer C (37°C, 30 min). Probe (GenePharma Corporation, Shanghai, China) hybridization was performed overnight at 37°C in dark using 100μL hybridization mixture containing: (1) denatured probes (100μM stock prepared in DEPC water, denatured at 75°C for 10 min), (2) SA-Cy3 conjugate, and (3) Buffer E (pre-warmed at 73°C until clarification) at 2:1:7 ratio. Post-hybridization washes included: 0.1% Buffer F (42°C, 5 min), 2× Buffer C (5 min), and 1×Buffer C (5 min). Nuclei were counterstained with DAPI (1:1000 dilution in PBS, 15 min, dark) before mounting with antifade medium. Images were captured using an LSM 900 confocal microscope (Leica, Mannheim, Germany).

### Ribonuclease R trypsinization

About 2µg of total RNA samples was incubated with ribonuclease (RNase) R (Epicentre Technologies,Wisconsin, America) for 10 min at 37°C. Total RNA samples without any treatment were used as controls. qRT-PCR was used to determine the expression levels of Circ_0007552 and its parental genes RILPL1.

### Cell transfection

Circ_0007552 interference(sh-Circ_0007552) and overexpression (OE-Circ_0007552) lentivirus vectors and the corresponding negative control (NC) vectors (sh-NC and OE-NC), negative control mimics (mimics NC), negative control inhibitors (inhibitors NC), miR-7974 mimics and miR-7974 inhibitors were synthesized by GenePharma Co., Ltd (Shanghai, China). Transfection was conducted by Lipofectamine 2000 reagent (Invitrogen, Carlsbad, CA, USA) following the manufacturer’s instruction. The experimental steps for lentivirus infection are as follows, a 96-well plate is used to set up a viral gradient for the transfection of SPCA-1, H1299, H838, and PC-9 cells. The infection multiplicity (MOI) for SPCA-1 and H1299 is set at 40, while the optimal MOI for H838 and PC-9 is 50. A 1/2 volume transfection method is employed. The concentrations of the lentiviruses are LV-shRNA-NC (4×10^8 TU/ml), LV-shRNA-Circ_0007552 (8×10^8 TU/ml), LV-Vector (1×10^9 TU/ml), and LV-OE-Circ_0007552 (2×10^8 TU/ml). The cells in the 6-well plate are washed with PBS, and 1ml of fresh complete medium is added to each well. The amount of lentivirus stock solution is calculated using the formula [Virus volume per well (μL) = (MOI × cell count)/viral titer (TU/ml)] × 1000. Then, 2 μL of polybrene at a concentration of 5 ug/μL is added to each well. After 24 hours of lentivirus transfection, 2ml of fresh complete medium is added. Fluorescence inversion microscopy is performed 72 hours after transfection. After 96 hours of infection, the lung adenocarcinoma cell lines stably overexpressing or stably downregulating CIRC_0007552 are selected using puromycin.

### Quantitative real-time PCR

The total RNA of LUAD cells was extracted by Trizol reagent (Thermo Scientific, Waltham, MA, USA) following the manufacturer’s instruction, and RNA purity was evaluated by UV spectrophotometry (NanoDrop 2000, Thermo Scientific). The A260/A280 ratio was used to assess protein contamination (acceptable range:1.8-2.0). RNA used for cDNA synthesis was 500ng. cDNA was synthesized from RNA by PrimeScript™RT reagent Kit (Takara, Japan). The relative RNA expression was probed by the quantitative real-time (qRT-PCR), which was accomplished by SYBR^®^ Premix Ex Taq II 820A (Takara, Japan). Circ_0007552, miR-7974 and BAP1 mRNA expression levels were normalized by β-actin and U6. Primer sequences list in **Supplementary Table S1**. qRT-PCR detection was placed in the fluorescence quantitative PCR instrument (ABI 7500). The reaction program of miRNAs in the initial template denaturation was 95°C for 15 minutes and the template denaturation in the PCR cycle was 94°C for 20 seconds. The annealing and extension were 60°C for 34 seconds, and 40 cycles were set. The reaction program of Circ_0007552 and BAP1 was 95°C for 30 seconds and the template denaturation in the PCR cycle was 95°C for 5 seconds. The annealing and extension were 60°C for 34 seconds, and 40 cycles were set.

### Cell counting kit-8 assay

LUAD cells of were seeded in a 96-well plate (2000–3000 cells per well). After cell attachment, cell proliferation viability was examined using cell counting kit-8 (CCK-8) reagent (MCE, MedChemExpress, shanghai, China) once every 24h for 5 days. The cells were incubated with 10μl CCK-8 reagent in a 37°Cincubator for 2h. The absorbance of the reaction mixture at 450nm was measured(Bio-Rad, Hercules, CA, USA).

### Colony formation assay

Cells were seeded in 6-well plates at densities optimized for each cell line (typically 500 cells/well) and cultured in complete medium for 14 days. Colonies were fixed with 4% paraformaldehyde (15 min), stained with 0.5% crystal violet (30 min), and counted the numbers of clones manually under bright-field microscopy.

### Wound healing assay

Each 6-well plates was inoculated with about 1x10^6^ LUAD cells. On the 2nd day, after the cells were adhered to the wall, a vertical line was drawn with a 200µl tip, the cells were washed with PBS for 2 times, and then added with 2ml of serum-free culture medium to continue the incubation. The width of intersection of the scratches was photographed and recorded at 0h, 24h, 48h and 72h respectively. Image J was used to compute the cells’ relative migration rate.

### Transwell invasion and migration assay

To evaluate invasive capacity, seeded tumor cells (4×10^4^ cells/well) in serum-free medium into Matrigel-coated (1:8 dilution) Transwell inserts (8-µm pores, Corning). The lower chamber contained complete medium with 10% FBS as chemoattractant. After 24h incubation (37°C, 5% CO_2_), non-invading cells were removed from the upper surface with cotton swabs. Invaded cells on the lower membrane were fixed with 4% paraformaldehyde at room temperature for 30 minutes, stained with 0.1% crystal violet for 15 minutes, and quantified by counting five random fields per insert under phase-contrast microscopy (200×magnification). Three independent experiments were performed. The steps of Transwell migration assay were the same as above, except that the chambers were not coated with Matrigel.

### Western blot

Proteins were extracted from cells and tissues using RIPA lysis buffer (Servicebio, Wuhan, China) with PMSF(Add 5μL PMSF to each 1 mL lysate), and quantified using a bicinchoninic acid kit (Beyotime). Protein of Samples were 20μg mixed with loading buffer, boiled at 100°C for 5 min, and then separated on a 12% polyacrylamide gel and transferred onto a polyvinylidene fluoride (PVDF) membrane (Millipore, USA, 0.22μm). Then, the PVDF membranes were blocked with 5% skimmed milk for 1 h and then incubated with primary antibodies at 4°Cfor 2h. After four washes with TBST, the membranes were incubated with horseradish peroxidase-labeled secondary antibody for 2 h. The protein blots were visualized by ECL-Plus reagent (Lanjieke, Beijing, China), with β-actin as a control. The antibodies were anti-BAP1 (Santa Cruz Biotechnology, USA, 1:500), anti-β-actin (Cell Signaling Technology, USA, 1:1000) and Goat Anti-Mouse IgG (Santa Cruz Biotechnology, USA, 1:3000).

### Dual-luciferase reporter gene assay

CircInteractome atabase (https://circinteractome.nia.nih.gov) and CircBank (https://www.circbank.cn) were used to predict the binding site between circ_0007552 and miR-7974. TargetScan Human 8.0 (https://www.targetscan.org) and miRDB (https://mirdb.org) were used to predict the binding sites Between Circ_0007552 and BAP1 wild-type (WT) and mutant type (MUT) vectors were constructed by GenePharma Corporation (Shanghai, China). 293T cells of 2x10^5^ were planted in 6-well plates for 24h and cotransfected with luc-empty vector, wild-type or mutant luc-circ-0007552 vector, and negative control or miR-7974 mimics, wild-type or mutant BAP1-UTR, and NC or miR-7974 mimics using Lipofectamine 2000 reagent (Invitrogen, Carlsbad, CA, USA). After 48h of co-transfection, the firefly fluorescence intensity and Renilla fluorescence intensity in each group were detected using dual-luciferase reporter kit (Biyuntian, China, RG027) according to the instructions.

### Animal studies

Four or five-week-old BALB/c nude mice were acquired from the Beijing Vital River Ltd., China, and maintained in a specific pathogen-free facility. The protocol was approved by the Institutional Animal Care and Use Committee of the Kunming Medical University Animal Center (approval number:Kmmu20220271). The experimental animals were randomly divided into four groups (sh-NC, sh-Circ_0007552, OE-NC and OE-Circ_0007552), five mice per group. The armpits of nude mice were injected with 4x10^5^ cells, and the tibial cavities was 2x10^5^. Every 5 days, the tumor’s growth was observed and measured. The tumor volume was determined using the formula V=1/2 (length) (width) (width). After 4 weeks, nude mice were sacrificed, X-rays were taken to evaluate tibial BM and bone destruction. While, tumors were removed, and weighed.

### Statistical analysis

All experiments were performed with three independent biological replicates, defined as cells processed in separate experimental batches. Statistical analyses were conducted across these biological replicates (n=3) to assess reproducibility of findings between fundamentally independent samples. SPSS 20.0 and GraphPad Prism 8.0 were used for statistical analysis. All values were expressed as mean ± standard deviation. Both the chi-squared test and the student’s t-test were used to compare the two groups. The relationship between Circ_0007552 expression and clinicopathological variables using the chi-squared test and Fisher’s exact test. At least three times each experiment was conducted, and a *P*<0.05 was considered statistically significant.

## Results

### The expression of Circ_000007552 is downregulated in LUAD and its correlation with clinicopathological features

**Figure 1:**
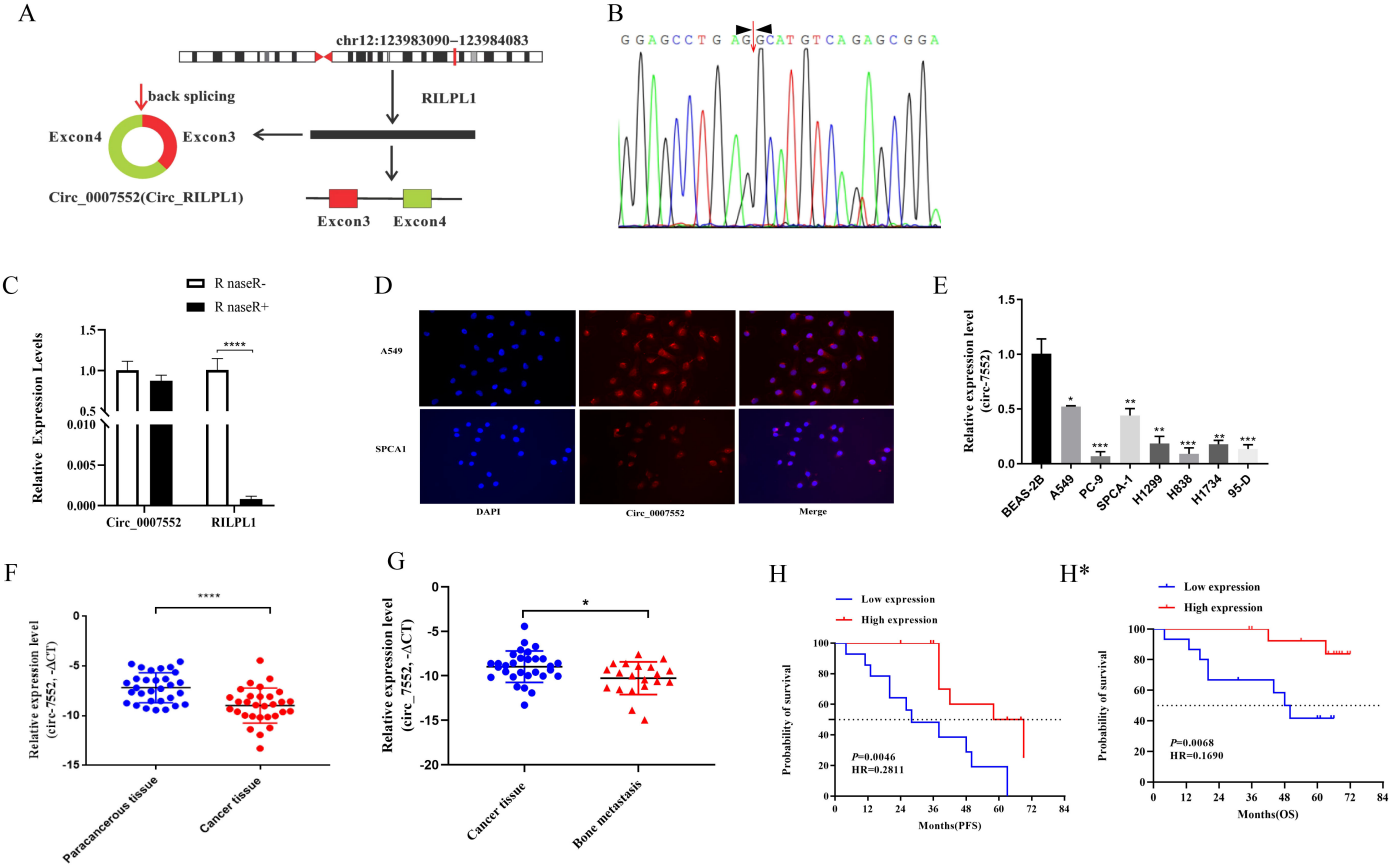
Low expression of Circ_0007552 in LUAD tissues and cells. **(A)** Genome localization and splicing pattern of Circ _ 0007552. **(B)** Identification of Circ _ 0007552 qPCR amplification products. Sanger sequencing of q PCR products (right). **(C)** The relative expression of Circ_0007552 and RILPL1 was detected by qRT - PCR after the total RNA of H1299 cells were incubated with RNase R. Statistical comparison by unpaired t-test confirmed this differential sensitivity. **(D)** RNA FISH detection showed that Circ _ 0007552 was mainly distributed in the cytoplasm(Magnification: x400). **(E)** Circ_0007552 expression in normal lung epithelial cells BEAS-2B and seven human LUAD cell lines (A549, PC-9, SPCA1, H1299, H838, H1734 and 95-D) was detected by qRT-PCR. **(F, G)** Circ_0007552 expression in LUAD tissues and adjacent tissues of 30 patients and 20 unmatched bone metastasis tissues was detected by qRT-PCR. **(H)** The relationship between Circ_0007552 expression and progression-free survival in LUAD patients was analyzed using the Kaplan–Meier curve. (H*) The relationship between Circ_0007552 expression and overall survival of LUAD patients was analyzed using the Kaplan–Meier curve. *P < 0.05, **P < 0.01, ***P < 0.001 and ****P < 0.0001.

To identify novel circular RNAs (circRNAs) in lung adenocarcinoma (LUAD), we performed RNA-seq sequencing analysis on primary LUAD lesions and bone metastatic lesions in our preliminary study. The results revealed that Circ_0007552 was significantly downregulated in bone metastatic lesions compared to primary LUAD lesions. Hsa_circ_0007552, derived from exons 3 and 4 of the parental gene RILPL1 transcript located on chromosome 12 through backsplicing, consists of 332 nucleotides (**Figure 1A**). Subsequently, we designed reverse primer to amplify Circ_0007552 and its Parental gene RILPL1.The qPCR products were subjected to Sanger sequencing. By comparing the sequencing sequences with the gene sequence of Circ_0007552, it was found that reverse splicing sites existed in the PCR products amplified by different primers (**Figure 1B**), confirming that Circ_0007552 existed in the form of a circular RNA in lung adenocarcinoma tissues. To further confirm the stability of Circ_0007552 expression, the RNase R degradation experiment results showed that RNase R treatment degraded the linear transcript of RILPL1, while Circ_0007552 was resistant to this treatment (**Figure 1C**). The RNA FISH detection results showed that Circ_0007552 was simultaneously distributed in the nucleus and cytoplasm of LUAD cells, but mainly in the cytoplasm (**Figure 1D**). In conclusion, these results indicate that Circ_0007552 is a newly discovered circular RNA in lung adenocarcinoma.

Then, The expression of Circ_0007552 in human LUAD cells and tissues was subsequently examined. A significant downregulation of Circ_0007552 expression was observed in tumor cells compared with normal lung epithelial cells through qRT-PCR analysis (**Figure 1E**). Furthermore, Circ_0007552 expression was detected in 30 paired tumor and adjacent normal tissues from non-metastatic lung adenocarcinoma patients. we used -△CT statistical methods to calculate the relative expression levels of Circ_0007552 in lung adenocarcinoma tissues. For skewed data, the median was used as the cut-off value. Thirty patients were divided into low-expression and high-expression groups, and further analysis was conducted on the relationship between Circ_0007552 and the patients’ clinical and pathological characteristics. Results revealing marked downregulation in tumor tissues relative to their normal counterparts (**Figure 1F**). This reduced expression level was found to be inversely correlated with tumor size and distant metastasis (**Supplementary Table S2**). Comparative analysis was performed on 20 non-matched bone metastasis samples, where Circ_0007552 expression was demonstrated to be further decreased in metastatic lesions compared to primary tumor tissues (**Figure 1G**). Moreover, Survival analysis revealed that patients exhibiting lower Circ_0007552 expression levels were associated with significantly reduced survival durations when compared to those with higher expression levels (Figure 1H, H*). Collectively, these findings indicate that Circ_0007552 is underexpressed in LUAD, with its downregulation serving as a prognostic indicator of unfavorable clinical outcomes.

### Circ_0007552 inhibited the viability, migration and aggressiveness of LUAD cells *in vitro*

To elucidate whether the differential expression of Circ_0007552 plays a functional role in lung cancer, the expression profile of Circ_0007552 was systematically examined in normal lung epithelial cells (BEAS-2B) and LUAD cells lines (A549, PC-9, SPCA1, H1299, H838, H1734, 95-D). Significant downregulation was consistently observed in all tested LUAD cells lines compared to normal controls, with particularly lower expression levels detected in PC-9 and H838 cells.

**Figure 2:**
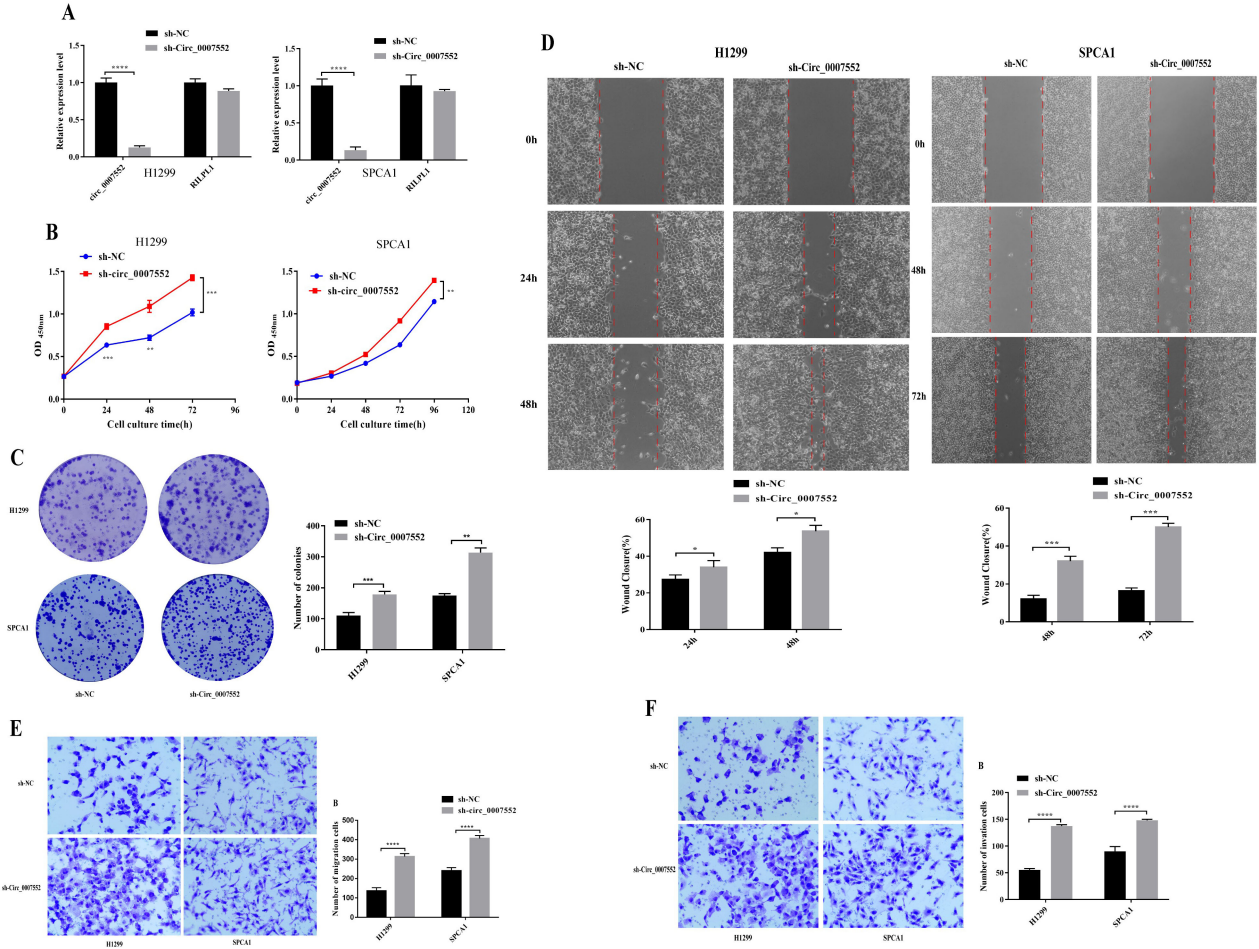
The impact of Circ_0007552 knockdown on the growth, migration and aggressiveness of LUAD cells. **(A)** Circ_0007552 expression in Circ_0007552 shRNA - transfected H1299 and SPCA1 cells was detected by qRT-PCR. **(B-F)** The proliferation, migration and invasion of Circ_0007552 knockdown LUAD cells were detected by CCK-8, colony formation, wound healing assays and transwell migration and invasion experiments. Magnification: x100, Statistical comparison by unpaired t-test confirmed this differential sensitivity. *P < 0.05, **P < 0.01, ***P < 0.001 and ****P < 0.0001.

**Figure 3:**
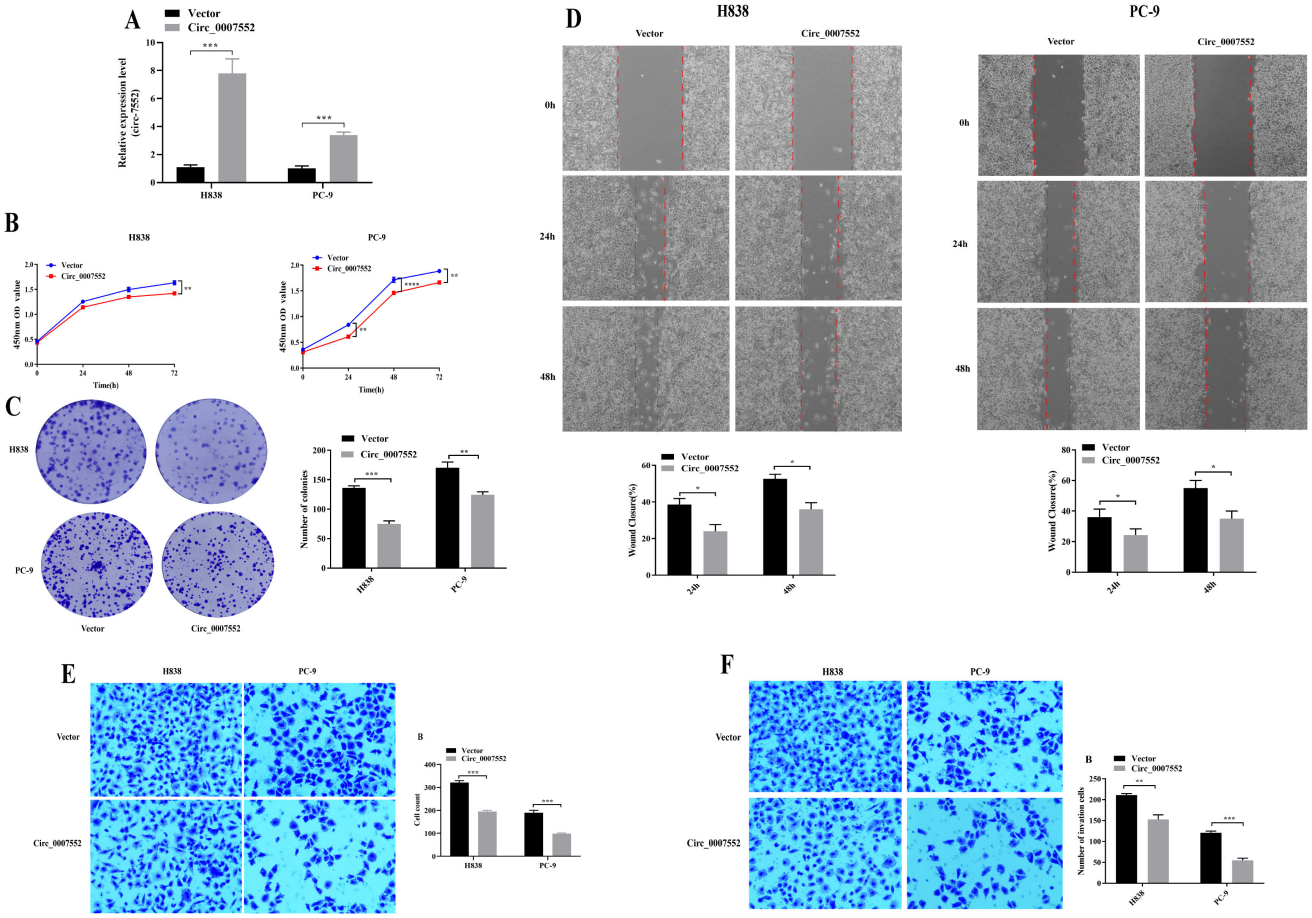
The effect of Circ_0007552 overexpression on the growth, migration and aggressiveness of LUAD cells. **(A)** Circ_0007552 expression in Circ_0007552 overexpressing lentivirus transfected H838 and pc-9 cells was detected by qRT - PCR. **(B-F)** The proliferation, migration and invasion of Circ_0007552 overexpressing LUAD cells were detected by CCK-8, colony formation, wound healing assays and transwell migration and invasion experiments. Magnification: x100, Statistical comparison by unpaired t-test confirmed this differential sensitivity. *P < 0.05, **P < 0.01, ***P < 0.001 and ****P < 0.0001.

To further investigate the role of Circ0007552 in LUAD cells, stable Circ0007552-overexpressing cell lines (Circ0007552) were constructed with H838 and PC-9 cells (**Figure 2A**), while Circ0007552-knockdown cell lines (sh-Circ_0007552) were established using H1299 and SPCA1 cells (**Figure 3A**), along with their respective negative controls (OE-NC and sh-NC). Fluorescent microscopy was employed to confirm successful lentiviral transfection by observing fluorescence signals, and the knockdown and overexpression efficiencies of Circ_0007552 were subsequently verified through qPCR analysis. Altered expression was specifically observed in Circ_0007552, with no significant changes detected in the parental gene expression.

To investigate the proliferative effects of Circ_0007552 in lung adenocarcinoma cells, CCK-8 assays and colony formation experiments were conducted. Compared with the lv3-control group, accelerated proliferative activity was observed in H1299 and SPCA1 cells following Circ_0007552 knockdown (**Figures 2B, C**), whereas suppressed proliferation was detected in Circ_0007552-overexpressing H838 and PC-9 cells relative to the OE-NC group (**Figures 3B, C**). The functional role of Circ_0007552 in cellular motility was further assessed through scratch wound healing assays, Transwell migration and invasion experiments. A significant enhancement in migratory capacity was demonstrated in Circ_0007552-knockdown H1299 and SPCA1 cells compared to the sh-NC group (**Figures 2D-F**), while inverse phenotypic outcomes were observed in Circ_0007552-overexpressing counterparts versus OE-NC controls (**Figures 3D-F**). Collectively, these data indicate that Circ_0007552, which was markedly downregulated across multiple lung adenocarcinoma cell lines, exerts inhibitory effects on malignant behaviors including proliferation, migration, and invasion.

### Suppression of Circ_0007552 expression inhibits subcutaneous tumor growth and bone metastasis *in vivo*

**Figure 4:**
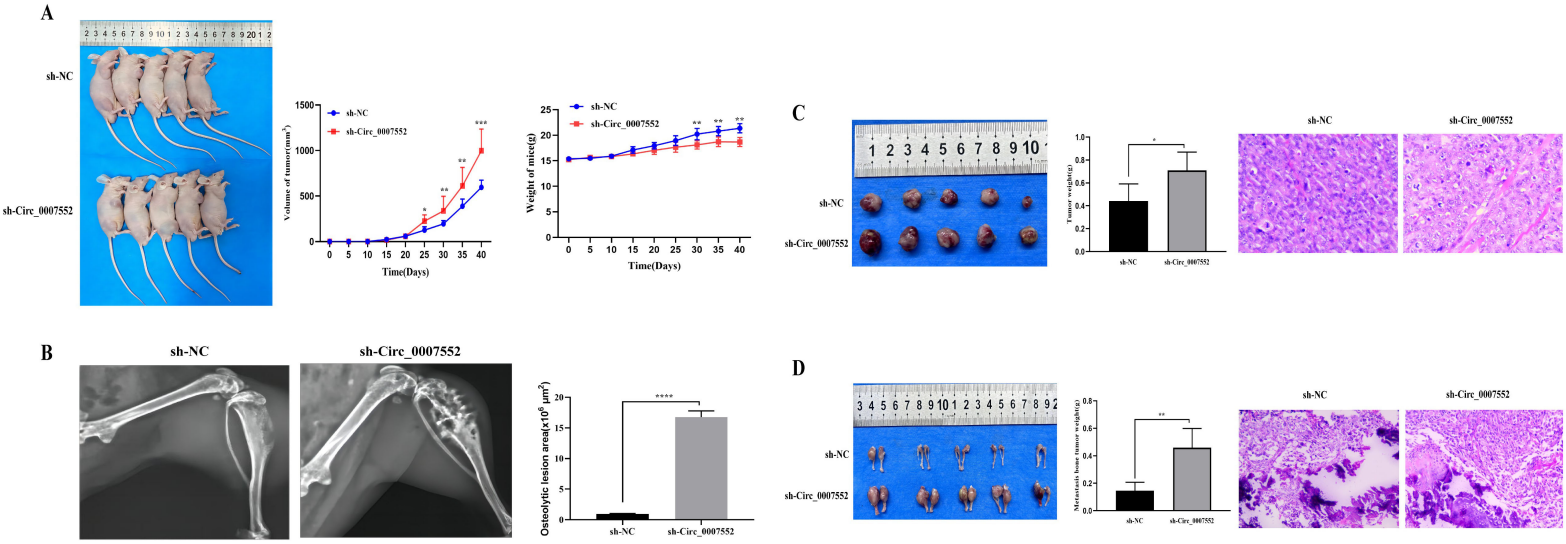
Circ_0007552 knockdown promotes the growth of implanted LUAD tumors in nude mice. Balb/C nude mice were implanted subcutaneously and in the femur with lentivirus-transduced ( n = 5 in each group ) sh-NC or sh-Circ _ 0007552 cells. **(A)** The dynamic growth of implanted tumors and weight of mice were monitored longitudinally. **(B)** BM areas were measured by X-ray. **(C)** The subcutaneous tumors were imaged to determine their size and excised for weight measurement. **(D)** The tumors in the femur were imaged to determine their size and excised for weight measurement. Magnification: x100, *P < 0.05, **P < 0.01 and ***P < 0.001.

To further elucidate the role and underlying mechanisms of Circ_0007552 in lung adenocarcinoma (LUAD) and bone metastasis (BM) in nude mice, H1299 and SPCA1 cells stably expressing sh-Circ_0007552 or sh-NC, as well as H838 and PC-9 cells stably transfected with Circ_0007552-overexpressing (OE-Circ_0007552) or negative control (OE-NC) vectors, were injected into the right axilla and bilateral tibiae of nude mice. It was observed that mice in the sh-Circ_0007552 group exhibited less weight gain compared to the sh-NC group, while their subcutaneous tumors demonstrated accelerated growth rates (**Figure 4A**). Upon reaching the study endpoint, both subcutaneous tumors and BM lesions harvested from the sh-Circ_0007552 group displayed significantly greater weights than those from the sh-NC group (**Figures 4C, D**). Furthermore, poorer differentiation of subcutaneous tumors was observed in sh-Circ_0007552 nude mice through hematoxylin-eosin staining. X-ray imaging revealed that the sh-Circ_0007552 group exhibited significantly elevated levels of BM invasiveness and bone destruction compared to controls (**Figures 4B, D**).

**Figure 5:**
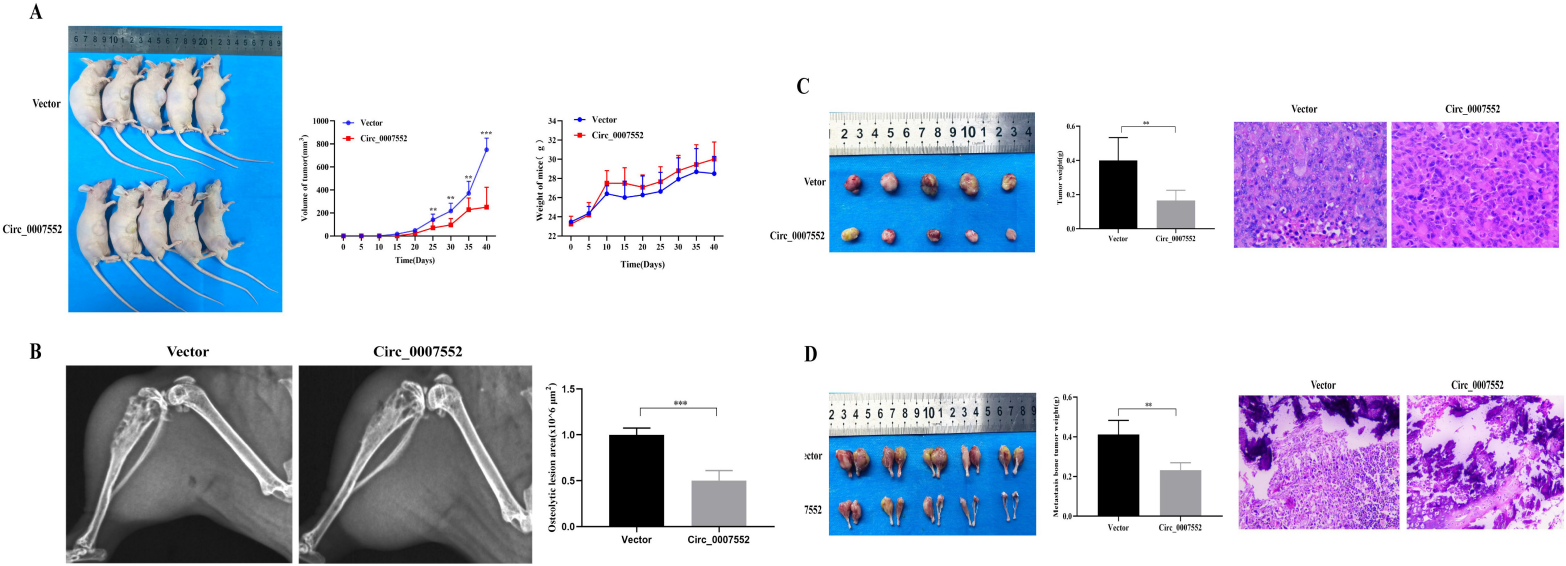
Circ_0007552 overexpression inhibits the growth of implanted LUAD tumors in nude mice. Balb/C nude mice were implanted subcutaneously and in the femur with lentivirus-transduced (n=5 in each group) Vector(OE-NC) or Circ_0007552 (OE-Circ_0007552) cells. **(A)** The dynamic growth of implanted tumors and weight of mice were monitored longitudinally. **(B)** BM areas were measured by X-ray. **(C)** The subcutaneous tumors were imaged to determine their size and excised for weight measurement. **(D)** The tumors in the femur were imaged to determine their size and excised for weight measurement. Magnification: x100, *P < 0.05, **P < 0.01 and ***P < 0.001.

In contrast, increased weight gain was recorded in OE-Circ_0007552 mice relative to the OE-NC group, accompanied by slower subcutaneous tumor growth (**Figure 5A**). At the experimental endpoint, both subcutaneous tumors and BM lesions harvested from the OE-Circ_0007552 group demonstrated reduced weights compared to those from the OE-NC group (**Figures 5C, D**). Additionally, improved tumor differentiation was identified in OE-Circ_0007552 nude mice via HE staining. X-ray imaging demonstrated that significantly reduced areas of BM invasion and bone destruction were observed in the OE-Circ_0007552 group compared to controls (**Figure 5B**). Collectively, these *in vivo* findings indicate that Circ_0007552 suppresses the growth and metastasis of lung adenocarcinoma cells.

### Circ_0007552 targeted miR-7974 in LUAD cells

**Figure 6:**
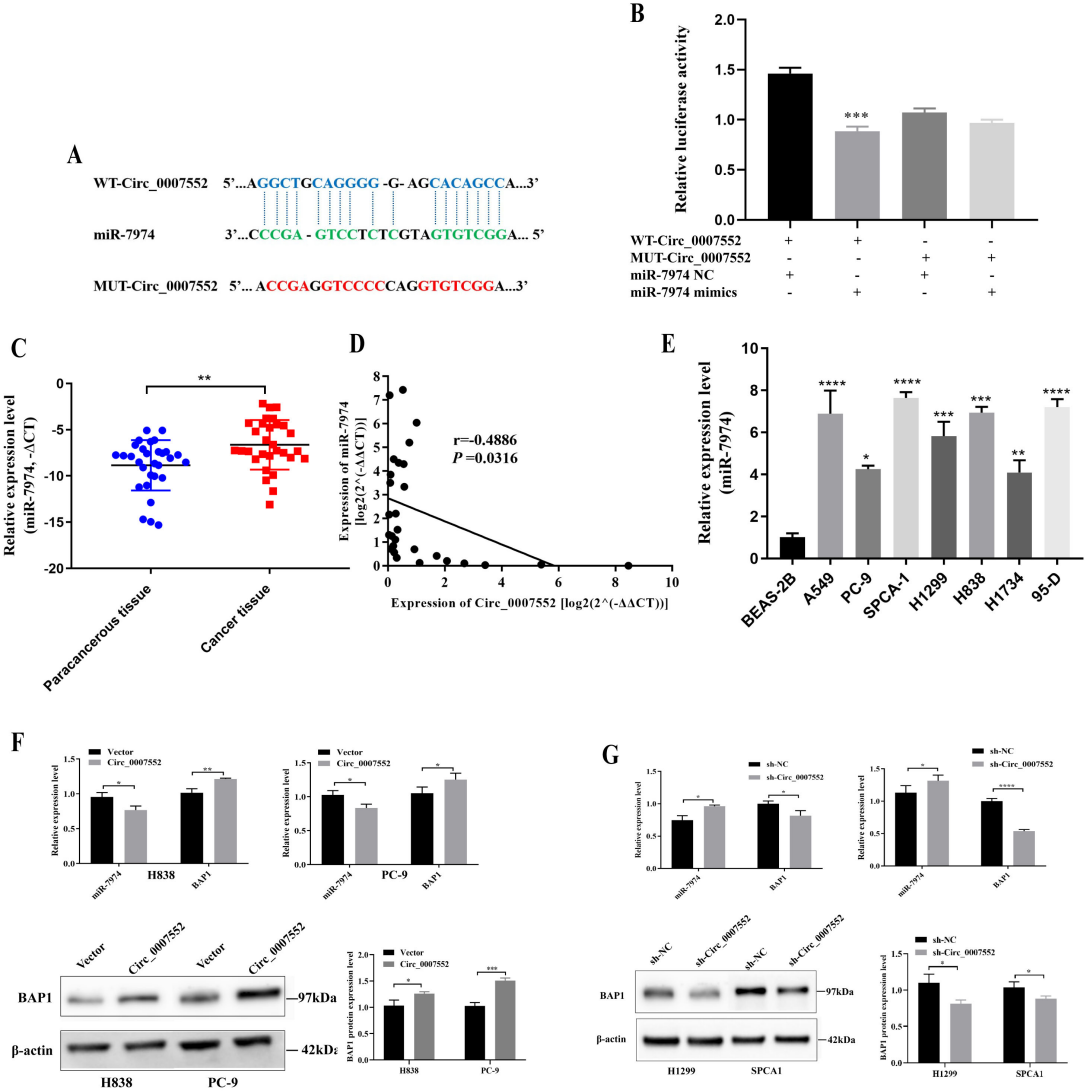
*miR-7974* was the target gene of *Circ_0007552* in LUAD cells. **(A)** The binding site between Circ _ 0007552 and miR-7974 was predicted using the CircInteractome database (https://circinteractome.nia.nih.gov/). **(B)** The binding relationship between *miR-7974* and C*irc _0007552* in 293T cells was verified using Dual - luciferase reporter gene assay. **(C)** *miR-7974* expression in LUAD tissues and adjacent tissues of 30 patients was detected by qRT-PCR. **(D)** The correlation between *Circ_0007552* and *miR-7974* expression levels in 30 LUAD tissues were evaluated by Pearson’s correlation analysis. **(E)** *miR-7974* expression in normal lung epithelial cells BEAS-2B and seven human LUAD cell lines (A549, PC-9, SPCA1, H1299, H838, H1734 and 95-D) was detected by qRT-PCR. **(F, G)** The expression of miR-7974 and BAP1 mRNA in LUAD cells with overexpression or knockdown of circ _ 0007552 was determined by qRT-PCR, and the expression of BAP1 protein was detected by Western blot. *P < 0.05, **P < 0.01, ***P < 0.001 and ****P < 0.0001.

Bioinformatic analysis revealed that a potential binding site between Circ_0007552 and miR-7974 was identified (**Figure 6A**). The high expression of miR-7974 in LUAD tissues was confirmed by qRT-PCR (**Figure 6C**). Furthermore, a negative correlation was observed between the expression of Circ_0007552 and miR-7974 in LUAD tissues (**Figure 6D**), suggesting a regulatory interaction. Additionally, miR-7974 was found to be upregulated in lung cancer cells compared to the normal pulmonary epithelial cell line BEAS-2B (**Figure 6E**). In dual-luciferase reporter assays, the activity of Circ_0007552 WT was significantly suppressed by miR-7974 mimics, whereas no effect was detected on Circ_0007552 MUT (**Figure 6B**). Notably, overexpression of Circ_0007552 in PC-9 and H838 cells was demonstrated to markedly inhibit miR-7974 expression, while knockdown of Circ_0007552 enhanced miR-7974 expression (**Figures 6F, G**). These findings collectively indicate that miR-7974 is directly targeted by Circ_0007552.

### Circ_0007552 suppresses the proliferation, migration, and invasion of LUAD cells via miR-7974

**Figure 7:**
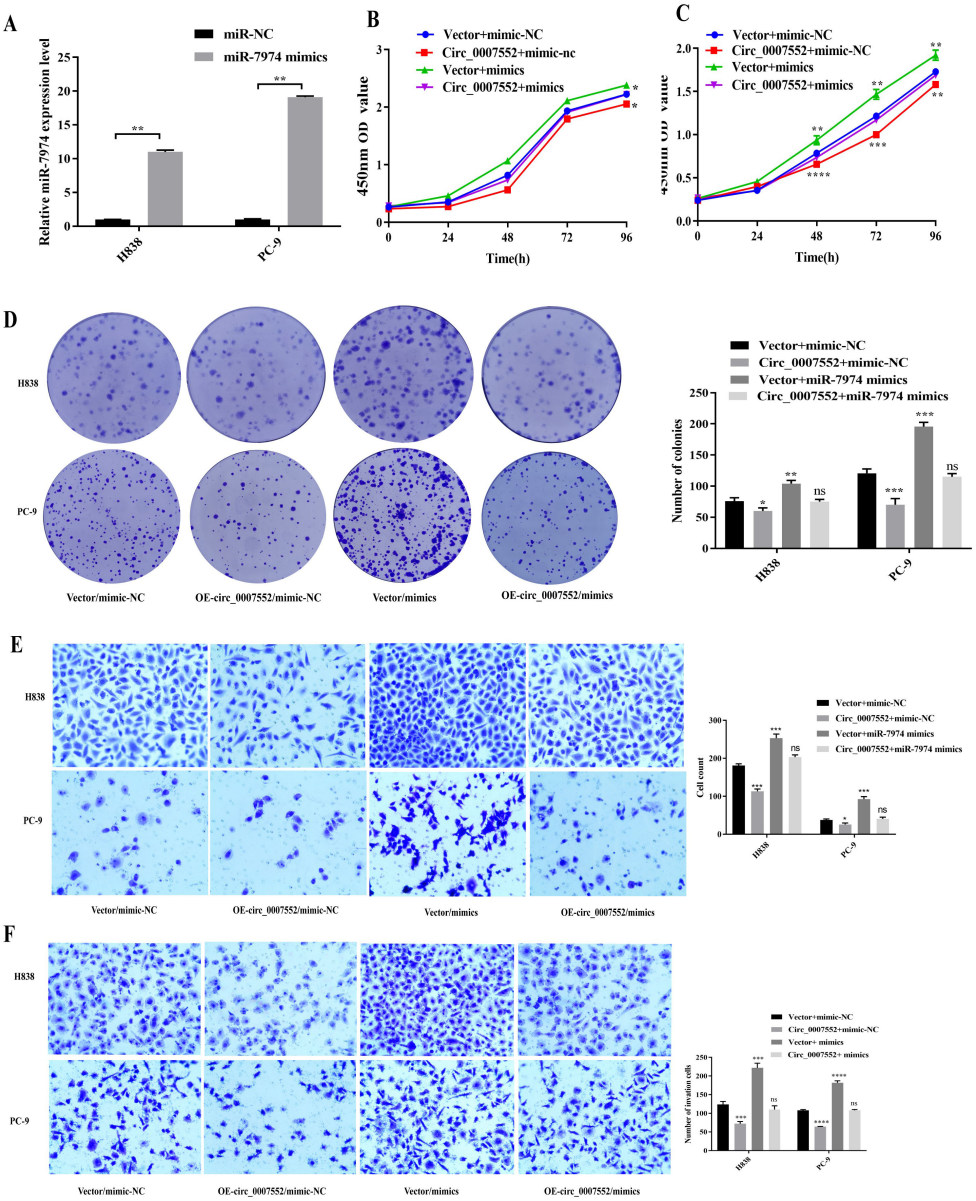
miR*-7974* rescued the impact of *Circ_0007552* on the growth, migration and invasion of LUAD cells. **(A)** miR-7974 expression in H838 and PC-9 cells transfected with miR-7974 mimics was detected by qRT - PCR. **(B–F)** Co-transfected with miR-7974 NC and mimics, H838 and PC-9 cells overexpressed Circ _ 0007552, and their growth, migration as well as invasion were detected by CCK-8, colony formation assay and transwell assay. Magnification: x100, *P < 0.05, **P < 0.01,***P < 0.001 and ****P < 0.0001.

To elucidate the mechanism of the Circ_0007552/miR-7974 axis in LUAD cells, miR-7974 mimics were transfected into Circ_0007552-overexpressing PC-9 and H838 cells (**Figure 7A**). Functional assessments including CCK-8, colony formation, and Transwell migration and invasion assays were subsequently performed. It was observed that miR-7974 overexpression partially rescued the inhibitory effects of Circ_0007552 overexpression on proliferative, migratory, and invasive capacities in both PC-9 and H838 cells (**Figures 7B-F**). These findings confirm that the biological behaviors of LUAD cells are regulated by Circ_0007552 through its sponging interaction with miR-7974.

### Circ_0007552 inhibits the proliferation and invasion of LUAD cells by regulating the miR-7974/BAP1 axis

**Figure 8:**
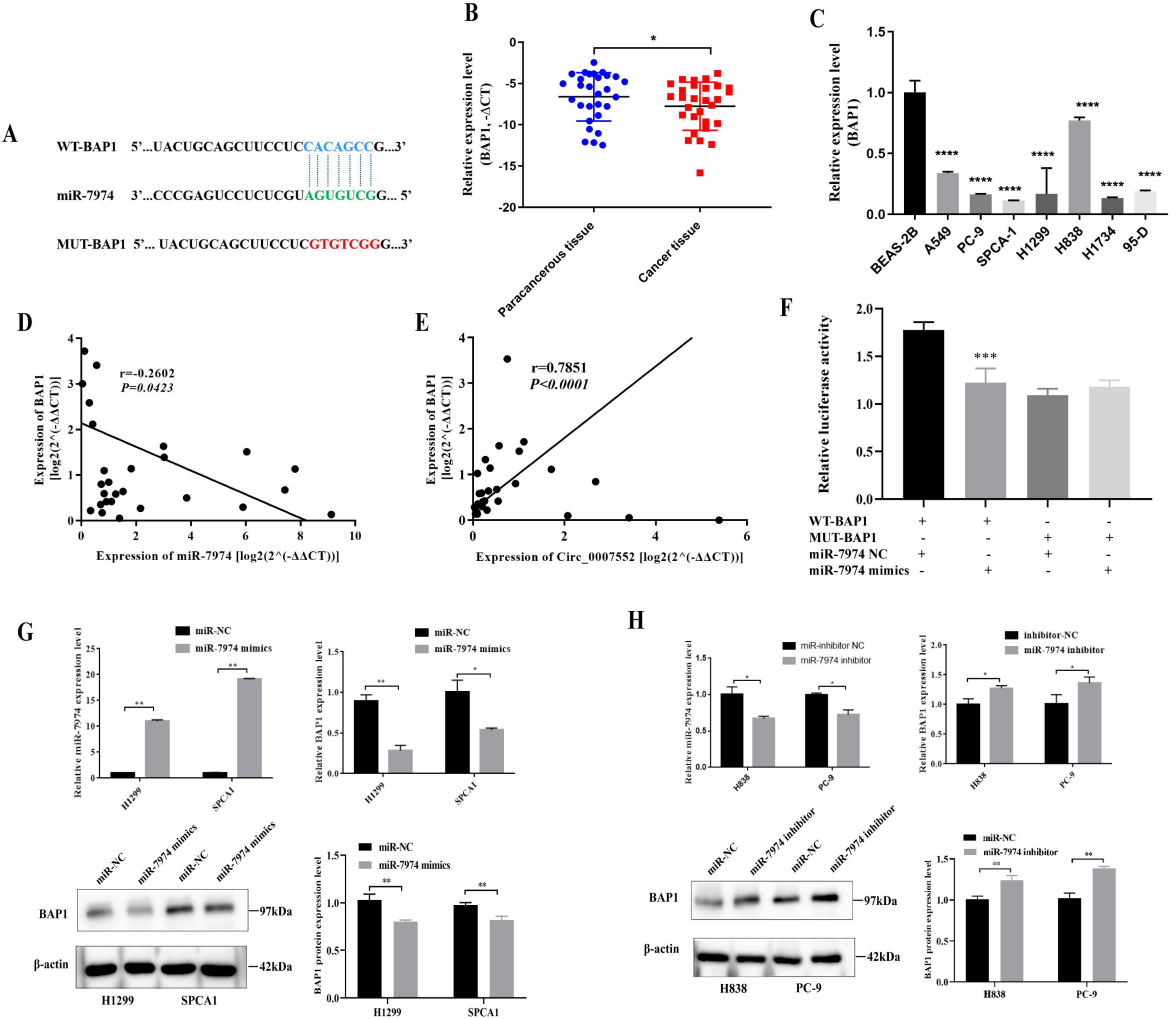
BAP1 was the target gene of miR-7974 in LUAD cells. **(A)** The binding site between miR-7974 and BAP1 3′UTR was predicted using TargetScan Human 8.0. **(B)** BAP1 mRNA expression in LUAD tissues and adjacent tissues of 30 patients was detected by qRT-PCR. **(C)** BAP1mRNA expression in normal lung epithelial cells BEAS-2B and seven human LUAD cell lines (A549, PC-9, SPCA1, H1299, H838, H1734 and 95-D) was detected using qRT-PCR. **(D, E)** The correlation between BAP1mRNA expression and miR-7974 expression, BAP1 mRNA expression and Circ _ 0007552 expression in 30 LUAD tissues were analyzed by Pearson’s correlation analysis. **(F)** The binding relationship between miR-7974 and BAP1 3′UTR in 293T cells was verified using Dual-luciferase reporter gene assay. **(G, H)** BAP1 protein expression in H1299 and SPCA1 cells transfected with miR-7974 mimics, H838 and PC-9 cells transfected with miR-7974 inhibitors was detected by Western blot. *P < 0.05, **P < 0.01, ***P < 0.001 and ****P < 0.0001.

Through in silico analysis utilizing the StarBase and miRDB database, BAP1 was identified as a potential target of miR-7974 (**Figure 8A**). qRT-PCR analysis revealed significantly reduced expression of BAP1 in both lung adenocarcinoma (LUAD) tissues and cell lines (**Figures 8B, C**). In Lung cancer tissues, BAP1 mRNA levels demonstrated an inverse correlation with miR-7974 expression but exhibited a positive correlation with circ_0007552 expression (**Figures 8D, E**). Dual-luciferase reporter assays demonstrated that miR-7974 significantly reduced the activity of BAP1-WT, whereas no pronounced effect was observed on BAP1-MUT (**Figure 8F**). Western blot analysis revealed that BAP1 protein expression was downregulated in lung adenocarcinoma cells following miR-7974 overexpression and Circ_0007552 knockdown. Furthermore, transfection with miR-7974 mimics was shown to partially reverse the regulatory effects of Circ_0007552 overexpression on BAP1 protein levels (**Figures 8G, H**), collectively indicating that BAP1 expression is modulated by the Circ_0007552/miR-7974 axis.

**Figure 9:**
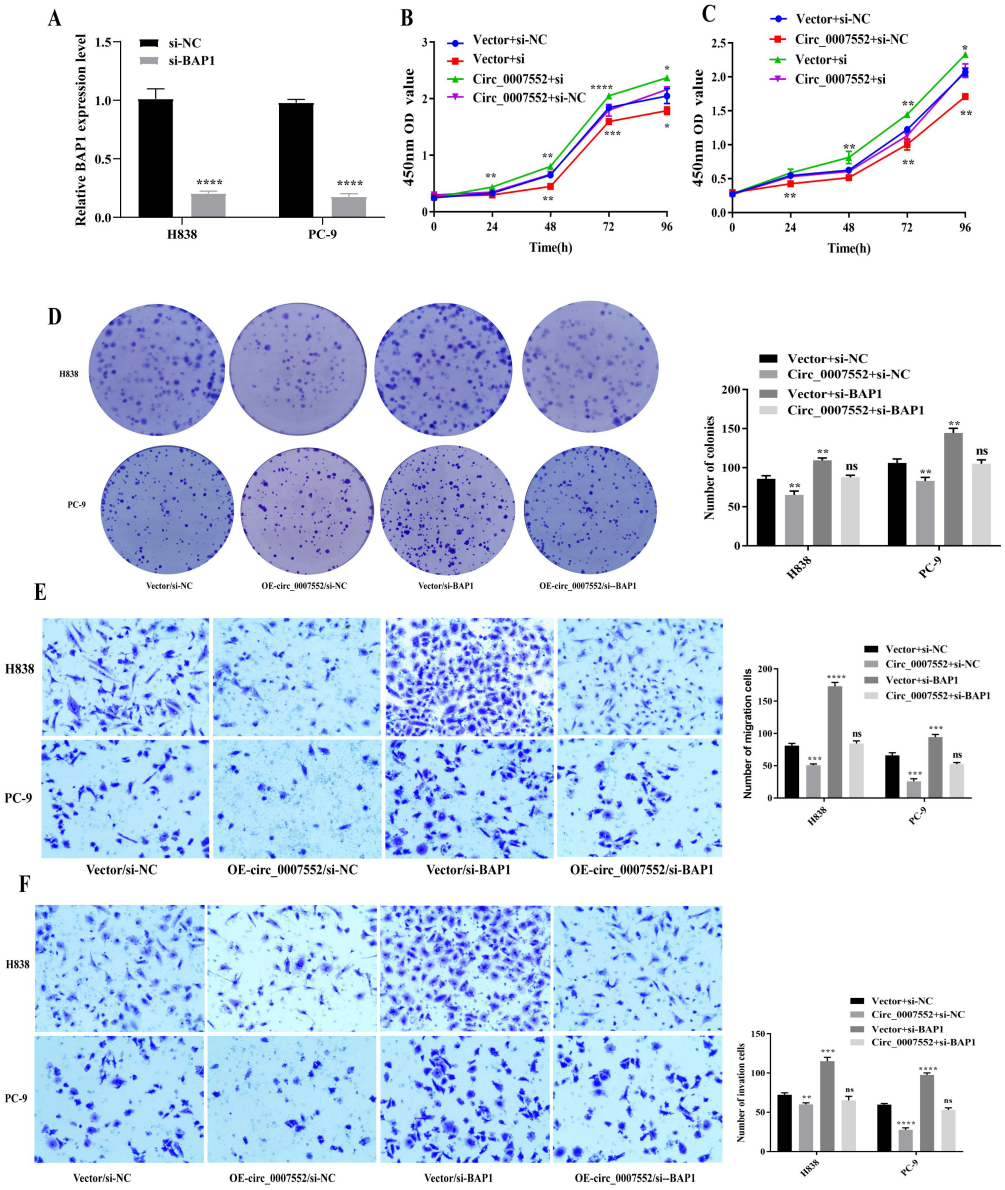
Suppressed BAP1 rescued the impact of *Circ_0007552* on the growth, migration and invasion of LUAD cells. **(A)** BAP1 knockdown in H838 and PC-9 cells with miR-7974 mimics was detected by qRT-PCR. **(B–F)** Co-transfected with si- NC and si-BAP, H838 overexpressed PC-9 cells overexpressed Circ_0007552, and their growth, migration and invasion were detected using CCK-8, clone formation assay and transwell assays. Magnification: x100, *P < 0.05, **P < 0.01,***P < 0.001 and ****P < 0.0001.

To further investigate whether BAP1 mediates the tumor-suppressive effects of Circ_0007552, BAP1 siRNA (si-BAP1) was transfected into Circ_0007552-overexpressing PC-9 and H838 cells. qRT-PCR and Western blot analyses revealed that Circ_0007552 overexpression upregulated BAP1 mRNA and protein levels in lung cancer cells, while si-BAP1 transfection attenuated this regulatory effect (**Figures 9A**). Functional assessments including CCK-8, colony formation, and Transwell migration and invasion assays demonstrated that BAP1 knockdown reversed the inhibitory effects of Circ_0007552 overexpression on lung cancer cell proliferation, migration, and invasion (**Figures 9B-F**).

## Discussion

circRNAs are characterized by high conservation, stability, and tissue-specific expression, making them promising candidates as molecular biomarkers for diagnosis, novel therapeutic targets, and prognostic predictors in lung adenocarcinoma, with potential clinical value in precision oncology research (8). With advancing investigations into circRNAs, researchers have revealed their ubiquitous presence in the human body and their involvement in diverse pathophysiological processes. Notably, significant progress has been made in understanding their roles in tumor biology (9). In multiple cancer studies, newly identified circRNAs have been continuously characterized and found to be closely associated with tumor progression and metastasis.

LU et al. (10) demonstrated that circCSNK1G3 promotes lung cancer progression by acting on the miR-143-3p/HOXA10 signaling axis. Another study (11) revealed that circSCAP directly binds to SF3A3 and facilitates its degradation, leading to elevated MDM4S levels that activate p53 signaling, thereby suppressing the occurrence and metastasis of NSCLC. ZHANG et al. (12) discovered that circRNA_010763 functions as a ceRNA by sponging miR-715, upregulating c-Myc expression and consequently promoting cancer cell proliferation, migration, and invasion. Additional studies (13) identified that the baseline separation of circRNA and its linear isomers can be achieved by optimizing chromatographic conditions, revealing the unique degradation pattern of circRNA, providing a new tool for vaccine stability assessment, and having important guiding significance for the development of circRNA therapeutic products.

Circ_0007552, a recently identified circular RNA derived from the parental gene *RILPL1* (associated with neuromuscular system disorders) (14) has garnered limited research attention to date. Current literature documents only one tumor-related study (15) reporting that circRILPL1 promotes malignant progression in nasopharyngeal carcinoma through activation of the Hippo-YAP signaling pathway. However, the potential role and molecular mechanisms of Circ_0007552 in LUAD remain unexplored and warrant systematic investigation. In this study, we observed significant downregulation of Circ_0007552 expression in LUAD tissues. Clinically, low Circ_0007552 expression correlated with advanced TNM staging, lymph node metastasis, and unfavorable prognosis in LUAD patients. Functional experiments demonstrated that Circ_0007552 overexpression suppressed cellular proliferation, migration, and invasion in lung cancer cells, whereas its knockdown exerted opposing oncogenic effects, suggesting its tumor-suppressive role in LUAD pathogenesis. Notably, these findings exhibit partial discrepancies with previous reports regarding circRILPL1’s oncogenic function in nasopharyngeal carcinoma, highlighting the necessity for further multi-cancer investigations to elucidate the context-dependent regulatory mechanisms of this circRNA.

To date, thousands of miRNAs have been identified in human cells as participants in the regulatory networks of cellular expression. Although the functional mechanisms of all miRNAs have not been fully elucidated, it has been established that miRNAs recognize and bind to complementary sites within the 3’-untranslated region (3’-UTR) of target gene mRNAs. This interaction leads to mRNA degradation and/or translational repression, thereby inhibiting target gene expression at both transcriptional and post-transcriptional levels (16). miR-7974, a newly discovered miRNA, has been reported to exhibit aberrant expression in viral infectious diseases (17) and osteonecrosis of the femoral head (18), suggesting its potential involvement in regulating biological processes such as cell proliferation and differentiation. However, research on miR-7974 in malignant tumors remains limited. The specific role of miR-7974 in LUAD has not been clearly defined. This study demonstrates that miR-7974 is overexpressed in LUAD cells and significantly promotes their proliferation, migration, and invasion, while its inhibition produces opposing effects. Consistent with our findings, Sexton et al. (19) identified miR-7974 as an oncogenic factor that facilitates the development and metastasis of gastric cancer. Emerging evidence has revealed that circular RNAs (circRNAs) possess diverse biological functions, including serving as miRNA sponges (20), interacting with RNA-binding proteins (21), regulating transcription (22), and encoding proteins (23). Our study corroborates that miR-7974 and Circ_0007552 share a binding site, with miR-7974 partially counteracting the biological effects of Circ_0007552 in LUAD cells. Notably, Circ_0007552 has been identified for the first time as a sponge molecule for miR-7974.

BAP1 was first identified and isolated from breast cancer and lung cancer cell lines in 1998, named for its binding to the RING domain of the BRCA1 protein. The BAP1-encoded protein functions as a deubiquitinating enzyme (24) that regulates protein stability, activity, and localization through deubiquitination modification of various substrate proteins. It plays extensive roles in cellular processes including gene expression regulation, DNA damage repair, cell cycle control, cellular metabolism, and programmed cell death (25). Initial studies suggested that BAP1 primarily localizes to the nucleus, where it interacts with BRCA1 to enhance its tumor-suppressive function (24). BAP1 is crucial for maintaining genomic stability and cellular signaling through its involvement in gene expression modulation, cell cycle regulation, and DNA repair mechanisms (26–28). Recent investigations have revealed that BAP1 can also localize to the cytoplasm, where it modulates calcium signaling and cell death pathways. Cytoplasmic BAP1 binds to IP3R3 and promotes calcium ion release from the endoplasmic reticulum into the cytosol via IP3R3 deubiquitination, thereby inducing apoptosis. Cells lacking BAP1 exhibit elevated apoptotic thresholds and increased susceptibility to malignant transformation (29). These findings underscore the essential role of intact BAP1 structure and deubiquitination activity in maintaining normal cellular functions, establishing BAP1 as a critical tumor suppressor. Although traditionally recognized as a tumor suppressor, emerging evidence indicates oncogenic roles of *BAP1* in certain malignancies. For instance BAP1 deubiquitination modified the transcription factor KLF5 in breast cancer cells promotes the growth of tumor cells, and the proliferation and invasion of tumor cells are weakened after BAP1 knocking down (30). Such findings suggest BAP1 may possess dual regulatory functions across different tumor types. As a critical tumor suppressor gene, mutational inactivation of BAP1 can lead to “BAP1 cancer syndrome” and the development of various sporadic tumors (31). Recent studies have revealed that BAP1 exhibits oncogenic functions in malignancies such as breast cancer and myeloid leukemia, which are associated with its biological activities.

Through this study, we demonstrated that BAP1 serves as a target gene of miR-7974 in LUAD. Furthermore, overexpression of Circ_0007552 or inhibition of miR-7974 significantly upregulated BAP1 expression, whereas knockdown of Circ_0007552 or overexpression of miR-7974 markedly downregulated BAP1 expression. Additionally, BAP1 knockdown partially reversed the inhibitory effects of Circ_0007552 overexpression on LUAD cells proliferation, migration, and invasion. Therefore, we conclude that Circ_0007552 suppresses the initiation, progression, and metastasis of lung cancer cells through the miR-7974/BAP1 axis. Notably, previous research (16) has reported that BAP1 regulates H2Aub deubiquitination at the SLC7A11 promoter, thereby suppressing SLC7A11 expression, inhibiting cystine uptake, and ultimately promoting ferroptosis. Given that ferroptosis represents a cutting-edge research frontier in oncology, future studies could focus on whether Circ_0007552 participates in ferroptosis regulation, either directly or indirectly. This scientific question merits further investigation to elucidate the underlying mechanisms, thereby providing a foundation for comprehensive mechanistic exploration.

## Conclusions

This study revealed that Circ_0007552 is downregulated in lung cancer tissues and cells, and its low expression correlates with clinicopathological characteristics in lung cancer patients. Furthermore, we demonstrated that Circ_0007552 suppresses the initiation, progression, and metastasis of lung cancer cells through the miR-7974/BAP1 axis, providing novel insights into the molecular mechanisms driving lung cancer advancement.

## Data Availability

The original contributions presented in the study are included in the article/**Supplementary Material**. Further inquiries can be directed to the corresponding author.
