# Supplementary material for: Circular RNA hsa_Circ_0007552 inhibits lung adenocarcinoma proliferation, migration and invasion via the miR-7974/BAP1 axis
Source: Front Immunol. 2025 Aug 21;16:1634326. doi: 10.3389/fimmu.2025.1634326 (PMC12408266; doi:10.3389/fimmu.2025.1634326)
Supplement: Supplementary file 1 [file Table1.docx]

**Supplementary Table S1. Sequences of primers**

| **Genes** |  | **Sequences（5'→3'）** |
| --- | --- | --- |
| Circ_0007552 | Forward Primer | CAGACCAAGGAGCAGGAGATG |
|  | Reverse Primer | CCGCTCTGACATGCCTCAG |
| RILPL1 | Forward Primer | CAAACAACGCGACGAGATCC |
|  | Reverse Primer | TGTTCGATCAGGGCTTTCCC |
| miR-7974 | Forward Primer | AGGCTGTGATGCTCTCCTGAG |
|  | Reverse Primer | Universal primers in the kit |
| miR-7156-3p | Forward Primer | CTGCAGCCACTTGGGGAAC |
|  | Reverse Primer | Universal primers in the kit |
| miR-766-3p | Forward Primer | ACTCCAGCCCCACAGCCTC |
|  | Reverse Primer | Universal primers in the kit |
| GAPDH | Forward Primer | TGCACCACCAACTGCTTAGC |
|  | Reverse Primer | GGCATGGACTGTGGTCATGAG |
| BAP1 | Forward Primer | ACCCTGCTCGTGGAAGATTT |
|  | Reverse Primer | AAGGTAGAGACCTTTCGCCG |
| U6 | Forward Primer | AAAGCAAATCATCGGACGACC |
|  | Reverse Primer | GTACAACACATTGTTTCCTCGGA |
| β-actin | Forward Primer | CCTTCCTGGGCATGGAGTC |
|  | Reverse Primer | TGATCTTCATTGTGCTGGGTG |

**Supplementary Table S2. Relationship between expression level of Circ_0007552 and clinical characteristics in lung adenocarcinoma**

| **clinical features** |  | **Total**  **n(%)** |  | **hsa_circ_0007552** | |  | ***P*** |
| --- | --- | --- | --- | --- | --- | --- | --- |
|  |  |  |  | **Low expression n(%)** | **High expression n(%)** |  |  |
| **Gender** |  |  |  |  |  |  | 0.419 |
| Male |  | 9(30.00) |  | 7(77.78) | 2(22.22) |  |  |
| Female |  | 21(70.00) |  | 12(57.14) | 9(42.86) |  |  |
| **Ages** |  |  |  |  |  |  | 0.626 |
| ≤50 |  | 5(16.67) |  | 4(80.00) | 1(20.00) |  |  |
| ＞50 |  | 25(83.33) |  | 15(60.00) | 10(40.00) |  |  |
| **Nationality** |  |  |  |  |  |  | 0.466 |
| Han |  | 14(46.67) |  | 10(71.43) | 4(28.57) |  |  |
| Minority |  | 16(53.33) |  | 9(56.25) | 7(43.75) |  |  |
| **Smoking history** |  |  |  |  |  |  | 0.419 |
| No |  | 21(70.00) |  | 12(57.14) | 9(42.86) |  |  |
| Yes |  | 9(30.00) |  | 7(77.78) | 2(22.22) |  |  |
| **T Stages** |  |  |  |  |  |  | **0.023** |
| T1-2 |  | 19(63.33) |  | 9(47.37) | 10(52.63) |  |  |
| T3-4 |  | 11(36.67) |  | 10(90.91) | 1(9.09) |  |  |
| **N Stages** |  |  |  |  |  |  | **0.029** |
| N0 |  | 14(46.67) |  | 6(42.86) | 8(57.14) |  |  |
| N+ |  | 16(53.33) |  | 13(81.25) | 3(18.75) |  |  |
| **Clinical stages** |  |  |  |  |  |  | **0.002** |
| I-II |  | 19(63.33) |  | 8(42.11) | 11(57.89) |  |  |
| III-IV |  | 11(36.67) |  | 10(90.91) | 1(9.09) |  |  |

**Supplementary Table S3. List of chemicals**

| **Chemicals** | **Manufacturer** |
| --- | --- |
| Primers of Circ_0007552, RILPL1 and BAP1 | Qingke Biology Co, LTD (China) |
| Primers of miR-7974、miR-7156-3p、miR-766-3p | Qingke Biology Co, LTD (China) |
| Primers of GAPDH、U6 | Qingke Biology Co, LTD (China) |
| Fluorescent probe of Circ_0007552 | Shanghai Gema Gene, China |
| Lentiviruses of sh-NC/sh-Circ_0007552 | Shanghai Gema Gene, China |
| Lentiviruses of Vector/OE-Circ_0007552 | Shanghai Gema Gene, China |
| has-miR-7974 mimics/NC | Shanghai Gema Gene, China |
| has-miR-7974 inhibitor/NC | Shanghai Gema Gene, China |
| Wild-type vectors of hsa_Circ_0007552 | Shanghai Gema Gene, China |
| Mutant vectors of hsa_Circ_0007552 | Shanghai Gema Gene, China |
| Wild-type vectors of hsa_BAP1 | Shanghai Gema Gene, China |
| Mutant vectors of hsa_BAP1 | Shanghai Gema Gene, China |
| has-si-NC/has-si-BAP1 | Shanghai Gema Gene, China |

**Supplementary Table S4. Reagent information**

| **Name of reagent** | **Manufacturer** |
| --- | --- |
| RNA later | Sigma，America |
| Trizol | Thermo Scientific, USA |
| Chloroform | Xilong Chemical Industry，China |
| 75% ethanol | Beijing Lanjieke Technology Co, LTD (China) |
| Isopropanol | Shanghai Chemical Reagent Company(China) |
| DEPC water | Biosharp，China |
| RNA extraction kit | Biosharp，China |
| Phosphate buffer solution (PBS) | Biosharp，China |
| Fetal bovine serum | Gibco, USA |
| Penicillin streptomycin double antibody | Hyclone, USA |
| 0.25% trypsin digest | Gibco, USA |
| Serum-free cell cryopreservation | Suzhou Xinse Mei Biotechnology Co, LTD (China) |
| Dimethyl sulfoxide (DMSO) | Biosharp，China |
| PrimeScript™RT reagent Kit | Takara，Janpan |
| SYBR® Premix Ex Taq II 820A | Takara，Janpan |
| MiRcute enhanced miRNA cDNA first chain synthesis kit | Tiangeng Biochemical Technology (Beijing) Co, LTD (China) |
| MiRcute enhanced miRNA fluorescent quantitative detection kit | Tiangeng Biochemical Technology (Beijing) Co, LTD (China) |
| RNase R kit | Epicentre Technologies, USA |
| RPMI 1640 | Biosharp，China |
| Matrigel gel | BD Biosciences, USA |
| Paraformaldehyde | Biosharp，China |
| Crystal violet | Biosharp，China |
| Neutral gum | China Shanghai specimen model factory, China |
| FISH test kit | Shanghai Gema Gene, China |
| Lipofectamine 2000 | ThermoFisher Scientific, USA |
| Bifluorescent reporter gene detection kit | Shanghai Biyuntian Biotechnology Co, LTD (China) |
| RIPA | Shanghai Biyuntian Biotechnology Co, LTD (China) |
| PMSF | Shanghai Biyuntian Biotechnology Co, LTD (China) |
| BCA protein concentration determination kit | Shanghai Biyuntian Biotechnology Co, LTD (China) |
| SDS-PAGE gel rapid configuration kit | Shanghai Biyuntian Biotechnology Co, LTD (China) |
| 5×SDS-PAGE protein loading buffer | Shanghai Biyuntian Biotechnology Co, LTD (China) |
| Pre-stained protein marker | Shanghai Biyuntian Biotechnology Co, LTD (China) |
| 20×TBST | Beijing Lanjieke Technology Co, LTD (China) |
| Dried skimmed milk | Beijing Solabao Technology Co, LTD (China) |
| Carbinol | Sichuan Xilong Science Co, LTD (China) |
| Ultra-sensitive ECL | Beijing Lanjieke Technology Co, LTD (China) |

**Supplementary Table S5. Antibodies information**

| **Antibodies** | **Manufacturer** |
| --- | --- |
| Monoclonal antibody of β-actin | Santa Cruz Biotechnology, USA |
| Mouse monoclonal antibody of BAP1 | Santa Cruz Biotechnology, USA |
| Garlic enzyme labeled goat anti-rabbit IgG | Santa Cruz Biotechnology, USA |
| Garlic enzyme labeled goat anti-mouse IgG | Santa Cruz Biotechnology, USA |

**Supplementary Table S6. Sequences of Lentiviruses**

| Group | Sequences（5’- 3’） |
| --- | --- |
| sh-NC | TTCTCCGAACGTGTCACGT |
| sh-Circ_0007552 | AGCCTGAGGCATGTCAGAG |
| Vector | TTCTCCGAACGTGTCACGT |
| OE-Circ_0007552 | GCATGTCAGAGCGGGAGCGACAGGTGATGAAGAAGCTGAAGGAGGTGGTGGACAAACAACGCGACGAGATCCGCGCCAAGGACAGGGAGCTGGGCCTGAAAAATGAGGACGTTGAGGCTTTACAGCAGCAGCAGACACGGCTGATGAAGATCAACCATGACCTTCGGCACCGGGTCACGGTGGTGGAGGCCCAGGGGAAAGCCCTGATCGAACAGAAGGTGGAGCTGGAGGCAGACCTGCAGACCAAGGAGCAGGAGATGGGCAGCCTGCGAGCAGAGCTGGGGAAGTTGCGAGAGAGGCTGCAGGGGGAGCACAGCCAGAATGGGGAGGAGGAGCCTGAG |

**Supplementary Table S7. Sequences of dual fluorescent enzyme plasmid**

| **Genes** | | **Sequences（5'→3'）** |
| --- | --- | --- |
| hsa_Circ_0007552 | WT | gctggggaagttgcgagagaGGCTgCAGGGGGagCACAGCCagaatggggaggaggagcctgag |
|  | MUT | gctggggaagttgcgagagaCCGAgGTCCCCCagGTGTCGGagaatggggaggaggagcctgag |
| hsa-BAP1 | WT | cattccttccatcgtgccctgaggctgacacggcagatcagccccatagtgctcaggaggcagcatctggagttggggcacagcgaggtactgcagcttcctcCACAGCCggctgtggagcagcaggacctggcccttctgcctgggcagcagaatatatattttacctatcagagacatctatttttctgggctccggacagcaggatcaagacaacccgttggagcccctgtgttccagaggacctgatgccaaggggtaatgggcccagcagtgcctctggagcccaggccccaaCACAGCCccatggcctctgccagatggctttgaaaaaggtgatccaagcaggcccctttatctgtacatagtgactgagtggggggtgctggcaagtgtgg |
|  | MUT | cattccttccatcgtgccctgaggctgacacggcagatcagccccatagtgctcaggaggcagcatctggagttggggcacagcgaggtactgcagcttcctcGTGTCGGggctgtggagcagcaggacctggcccttctgcctgggcagcagaatatatattttacctatcagagacatctatttttctgggctccggacagcaggatcaagacaacccgttggagcccctgtgttccagaggacctgatgccaaggggtaatgggcccagcagtgcctctggagcccaggccccaaGTGTCGGccatggcctctgccagatggctttgaaaaaggtgatccaagcaggcccctttatctgtacatagtgactgagtggggggtgctggcaagtgtgg |

**Supplementary Table S8**. **Sequences of chemicals**

| **Genes** | **S(5’-3’)** | **AS （5’-3’）** |
| --- | --- | --- |
| mimics-NC | UUC UCC GAA CGU GUC ACG UTT | ACG UGA CAC GUU CGG AGA ATT |
| miR-7974 mimics | AGGCUGUGAUGCUCUCCUGAGCCC | GCUCAGGAGAGCAUCACAGCCUUU |
| Inhibitor-NC | CAGUACUUUUGUGUAGUACAA | / |
| has-miR-7974 inhibitor | CCCGAUCCUCUCGUAGUGUCGGA | / |
| si-NC | UUC UCC GAA CGU GUC ACG UTT | ACG UGA CAC GUU CGG AGA ATT |
| si-BAP1 | GAGGCUGAGAUUGCAAACUTT | AGUUUGCAAUCUCAGCCUCTT |

**Supplementary Table S9. Sequences of FISH probe**

| FISH Probe | sequence |
| --- | --- |
| 18S probe（5’-3’） | CTGCCTTCCTTGGATGTGGTAGCCGTTTC |
| NC probe（5’-3’） | TGCTTTGCACGGTAACGCCTGTTTT |
| hsa_Circ_0007552  probe（5’-3’） | ACATGCCTCAGGC+TCCTCCTCCCCTCTGACA+  TGCCTCAGGC+TCCTCCTTCCCGCTC+TGACATGCCTCAGGCT |
